# Tourniquet Practice Among Orthopaedic Surgeons in Saudi Arabia

**DOI:** 10.7759/cureus.45828

**Published:** 2023-09-23

**Authors:** Abdulmalik B Albaker, Ismail Almogbil, Abdulaziz F Alkheraiji, Abdullah H Alshahrani, Sultan K Alharbi, Ghada F AlSwaji, Razan M Alotaibi, Alanoud Alrashidi

**Affiliations:** 1 Department of Orthopaedics, College of Medicine, Majmaah University, Al Majma’ah, SAU; 2 Department of Surgery, Unaizah College of Medicine and Medical Sciences, Qassim University, Buraydah, SAU; 3 Department of Orthopaedics, Unaizah College of Medicine and Medical Sciences, Qassim University, Buraydah, SAU; 4 Department of Orthopaedics, College of Medicine, Imam Mohammad Ibn Saud Islamic University, Riyadh, SAU; 5 Department of Surgery, College of Medicine, Princess Nourah Bint Abdulrahman University, Riyadh, SAU; 6 Department of Orthopaedics, King Fahad Medical City, Riyadh, SAU

**Keywords:** saudi arabia, limb occlusion pressure, cuff pressure, orthopaedic resident, tourniquet

## Abstract

Introduction

This study aimed to evaluate the knowledge of tourniquet use among orthopedic surgeons in Saudi Arabia and assess the practical aspects of their use of tourniquets and the complications they have experienced in their practices.

Materials and methods

This cross-sectional study was conducted from December 2022 to February 2023. An online questionnaire was distributed among orthopedic surgeons and trainees in Saudi Arabia, and the surgeons’ knowledge of tourniquet use was assessed using 17 questions. To investigate tourniquet usage, the participants were divided into three groups: orthopedic residents, specialists, and consultants. An upper limb cuff pressure (CP) of 200 mmHg and a lower limb CP of 250 mmHg were chosen as the cut-off values, and the doctors’ choices were compared against literature recommendations using these measures.

Result

A total of 205 participants filled out the questionnaires; 130 residents, 15 consultants, and 60 specialists, with more males (175/205) than females responding. One hundred and twenty-one surgeons placed the cuff on patients by themselves, while 50 (24.3%) surgeons asked nurses for aid; 135 (65.6%) of them work in teaching hospitals, while 50 (24.3%) work in community hospitals. The incidence of post-tourniquet syndrome was unrelated to expertise (p=0.12).

Conclusion

When applied properly, tourniquets prevent excessive bleeding and keep the operative field clean during limb surgeries. This study aims to inspire the orthopedic community to reconsider long-held practices, especially regarding tourniquet pressure. The addition of ligature safety education to orthopedics training and outlining the settings and procedures for applying pressure should also be considered. The orthopedic community should set CP and process criteria to avoid complications. This study showed the importance of modifying the training of orthopedic residents to raise awareness and prevent unpleasant events from occurring.

## Introduction

The term “tourniquet” is derived from the French verb “tourner” (which means “to turn”) and was invented by Jean Louis Petit. In limb surgery, applying a tourniquet reliably creates a bloodless operative field, ensuring a high safety margin and simplifying the procedure [1]. More than 15,000 procedures each day in the modern surgical theatre involve the use of tourniquets of various kinds [2]. A new device, the silicone ring tourniquet, was introduced recently [3,4]. Unlike modern pneumatic tourniquets, which may be adjusted to the patient's comfort level, traditional non-pneumatic tourniquets have a fixed amount of pressure [5]. A circular pneumatic cuff that can be inflated with high-pressure air using a programmable pump is the basis for the tourniquet, a surgical instrument. Tourniquet application causes temporary ischemia at the surgical site during open or arthroscopic procedures on the upper and lower limbs, allowing surgeons a clearer view of the structures in the field and making it simpler to distinguish between anatomical structures [6]. Gloved fingers, a Penrose drain, a urinary catheter, and a rubber band can all be used to form a tourniquet around the digits. Whether a hemostat is necessary for securing a digital tourniquet needs to be clarified. Sterile gauze strips have also been used as an alternative to the traditional tourniquet [7].

Essential to achieving a bloodless operative field during extremities procedures, tourniquets allow surgeons to safely and clearly expose anatomical structures with less technical complexity and in less time [2,8,9]. Despite its benefits, tourniquet use may be unsafe because of systemic and local side effects including post-operative pain and oedema, chemical burns, nerve and muscle damage, systemic metabolic effects, thrombosis, pulmonary embolism, intraoperative breakthrough bleeding, and compartment syndrome [8,10-13]. The application of a tourniquet may worsen pain during and after surgery, necessitating deeper anaesthesia or the use of more analgesic drugs [14]. After surgery, excessive pain might delay wound healing and prolong hospital stay [15]. Complications can often be avoided by reducing the tourniquet's cuff pressure (CP) and inflation time (TIT) [12]. However, various recommendations and individual preferences based on experience exist because there is no clear definition of an absolute CP level in the literature [2,7-9,16-18].

The study aimed to investigate the understanding of tourniquet use among Saudi orthopaedics and evaluate the practical aspects of their use of tourniquets and the complications they experienced in their practices.

## Materials and methods

This cross-sectional study was conducted from December 2022 to February 2023 through an online questionnaire which was created using guidelines from the Association of Perioperative Registered Nurses (AORN) for the recommended practice of tourniquet application and was modified by Yalçınkaya et al. [19,20]. The ethical approval was obtained from the Majmaah University Research Ethics Committee (MUREC) with IRB number (MUREC-Aug.30/COM-2022/12-2).

All orthopaedic surgeons and trainees working in Saudi private or public hospitals were included in the study. The surgeon's knowledge of tourniquet uses and associated consequences was evaluated using a 17-question survey.

To get the highest percentage of responses per physician surveyed, the survey was designed to ensure that participants reflected on their current understanding of the issue as if it were a regular operating day [21]. The questionnaire was pretested on a sample of 20 participants and found to have good reliability and validity with Cronbach's alpha of 0.7.

The physicians were questioned about their current knowledge of tourniquet use, academic standing, duration of practice, and workplace. The survey contained questions about the type of tourniquet, pressure, setting, and time restrictions. All participants were asked to record any tourniquet-related issues they had experienced within two years prior to this study. To examine any variations in tourniquet usage, the participants were split into three groups: orthopaedic residents (Group 1), consultants (Group 2), and orthopaedic surgeons (Group 3). Cuff pressures (CPs) of 200 mmHg and 250 mmHg were chosen as the cut-off values for the upper and lower limbs, respectively. These metrics were used to assess how well the doctors' preferences aligned with the literature's recommendations.

Statistical Package for Social Sciences (SPSS) version 23.0 (IBM Corp., Armonk, NY) was used to analyze the data. Data are presented as frequencies and percentages. The Kolmogorov-Smirnov test was applied to find out the distribution of data. The Pearson chi-square test, the t-test, and Pearson's correlation test were used for comparing the continuous variables. The level of statistical significance was set at 0.05.

## Results

Two hundred and five responses were obtained through the survey, including 130 medical residents, 15 consultants, and 60 specialists. Most of the respondents 85.3% (175/205) were males, and 14.6% (30/205) were females. One hundred and twenty-one surgeons applied the cuff on patients by themselves, while 50 (24.3%) asked nurses for aid; 135 (65.8%) respondents work in teaching hospitals, while 50 (24.3%) work in a community hospital. A total of 77 (37.5%) participants answered that nitrogen and ambient air are the gases that must never be used in pneumatic tourniquets, while 128 (62.4%) of them responded that nitrous oxide and oxygen are the gases that must never be used in pneumatic tourniquets. A cuff overlap of 2-4 inches, 3-6 inches, 4-6 inches, and 6-8 inches was considered appropriate by 83 (40.4%), 46 (22.4%), 62 (30.2%), and 14 (6.8%) participants, respectively; 52 (40%), 32 (24.6%), 40 (30.7%), and 6 (46.1%) residents, respectively; 22 (36.6%), 12 (20%), 19 (31.6%), and 7 (11.6%) specialists, respectively; and 9 (60%), 2 (13.3%), 3 (20%), and 1 (6.6%) consultants, respectively, with statistical non-significance (Table 1).

**Table 1:** Association of tourniquets use knowledge with a level of expertise

Questions on the knowledge of participants with regard to tourniquet use	Total participants N=205, (%)	Residents N=130, (%)	Consultants N=15, (%)	Specialists N=60, (%)	Chi-square	p-value
What are the two gasses that must never be used in pneumatic tourniquets?					10.25	0.0059
Nitrogen and ambient air	77 (37.5%)	47 (36.1%)	10 (66.6%)	20 (33.3%)		
Nitric oxide and oxygen	128 (62.4%)	83 (63.8%)	5 (33.3%)	40 (66.6%)		
How much cuff overlap must be when applying it on the limb?					6.18	0.40
2 to 4 inches	83 (40.4%)	52 (40%)	9 (60%)	22 (36.6%)		
3 to 6 inches	46 (22.4%)	32 (24.6%)	2 (13.3%)	12 (20%)		
4 to 6 inches	62 (30.2%)	40 (30.7%)	3 (20%)	19 (31.6%)		
6 to 8 inches	14 (6.8%)	6 (46.1%)	1 (6.6%)	7 (11.6%)		
What will result if there is too much overlap of the cuff?					2.35	0.0001
It will reduce the safe tourniquet	65 (31.7%)	27 (20.7%)	4 (6.6%)	22 (36.6%)		
It will increase pressure resulting in the rolling/wrinkling of tissue beneath	94 (45.8%)	73 (56.1%)	7 (46.6%)	14 (23.3%)		
It will cause burns on the underlying skin	46 (22.4%)	20 (15.3%)	4 (6.6%)	24 (40%)		
Why is large cuff width preferred?					22.22	0.00018
It increases the safe tourniquet time	46 (22.4%)	24 (18.4%)	4 (6.6%)	22 (36.6%)		
It is more effective in producing a bloodless operative field	56 (27.3%)	43 (33%)	8 (53.3%)	5 (8.3%)		
It occludes blood flow at lower pressure	103 (50.2%)	68 (52.3%)	3 (20%)	33 (55%)		
At what part of a limb should the cuff be positioned?					6.37	0.17
Proximal to the incision on the bone	125 (60.9%)	78 (60%)	12 (80%)	35 (58.3%)		
Proximal to the incision on the joint	25 (12.1%)	12 (9.2%)	1(6.6%)	12 (20%)		
Distal to the incision on the bone	20 (9.7%)	16 (12.3%)	0 (0%)	4 (6.66%)		
Distal to the incision on the joint	35 (17%)	26 (20%)	2 (13.3%)	7 (11.6%)		

When asked which part of the limb was best for placing the cuff, 125 (60.9%) participants chose “proximal to the incision on the bone,” 35 (17%) of them chose “distal to the incision on the joint,” 25 (12.1%) chose “proximal to the incision on the joint,” and 20 (9.7%) chose “distal to the incision on the bone” (Table 1). The respondents’ knowledge of the effect of too much cuff overlap and the benefits of a large cuff width was statistically significant with a p-value of 0.0001 and 0.00018, respectively (Table 1).

The association of tourniquet pressure knowledge with the participant’s level of expertise was analyzed and found to be statistically significant (Table 2).

**Table 2:** Association of practices of participants with regard to level of expertise

Questions on the knowledge of participants with regard to tourniquet pressure	Total number of participants N=205 (%)	Residents N=130, (%)	Consultants N=15, (%)	Specialist N=60, (%)	Chi-square	p-value
Define limb occlusion pressure (LOP)					45.8	<0.01
In order to stop arterial blood flow into the extremity distal to the cuff, the lowest tourniquet pressure is needed	103 (50.2%)	50 (38.4%)	2 (13.3%)	51 (85%)		
In order to stop arterial blood flow into the extremity distal to the cuff, the maximum tourniquet pressure is needed.	10 (4.8%)	4 (3%)	6 (40%)	0 (0%)		
To stop arterial blood flow into the extremity proximal to the cuff, the lowest tourniquet pressure is needed.	50 (24.3%)	42 (32.3%)	1 (6.6%)	7 (11.6%)		
To stop arterial blood flow into the extremities proximal to the cuff, the maximum tourniquet pressure is needed.	6 (2.9%)	4 (3%)	1 (6.6%)	1 (1.6%)		
I don’t know	36 (17.5%)	30 (23%)	5 (33.3%)	1 (1.6%)		
What cuff shape occludes arterial flow at lower pressure?					9.83	0.007
Curved (contoured) cuffs	95 (46.3%)	50 (38.4%)	9 (60%)	36 (60%)		
Straight (rectangular) cuffs	110 (53.6%)	80 (61.5%)	5 (33.3%)	24 (40%)		
What are two contraindications to pneumatic tourniquet use?					17.40	0.0079
Peripheral vascular disease, Debridement of open fracture	70 (34.1%)	30 (23%)	5 (33.3%)	40 (66.6%)		
Peripheral vascular disease	25 (12.1%)	21 (16.15%)	1 (6.6%)	3 (5%)		
Debridement of open fracture	10 (4.8%)	5 (3.8%)	0 (0%)	5 (8.3%)		
Peripheral vascular disease, Hypertensive patient	26 (12.6%)	15 (11.5%)	3 (20%)	8 (13.3%)		
Peripheral vascular disease, Hyperthyroid patient	20 (9.7%)	20 (15.3%)	0 (0%)	0 (0%)		
Hypertensive patient	12 (5.8%)	8 (6.15%)	1 (6.6%)	4 (6.6%)		
Hypertensive patient, Hyperthyroid patient, Debridement of open fracture	3 (1.4%)	2 (1.5%)	1 (6.6%)	0 (0%)		
Peripheral vascular disease, Hypertensive patient, Hyperthyroid patient, Debridement of open fracture	3 (1.4%)	2 (1.5%)	1 (6.6%)	0 (0%)		
Hypertensive patient, Hyperthyroid patient	20 (9.7%)	12 (9.2%)	0 (0%)	0 (0%)		
Hypertensive patient, Debridement of open fracture	6 (2.9%)	6 (4.6%)	0 (0%)	0 (0%)		
Hyperthyroid patient	6 (2.9%)	4 (3%)	2 (13.3%)	0 (0%)		
Hyperthyroid patient, Debridement of open fracture	4 (1.9%)	3 (2.3%)	1 (6.6%)	0 (0%)		
If a tourniquet has been applied & inflated but needs re-positioning before the operation starts, How should it be re-positioned?					8.27	0.015
The cuff underlying padding should be removed, new padding placed and the cuff reapplied	148 (72.1%)	85 (65.3%)	13 (86.6%)	50 (83.3%)		
Pull the cuff up and down and re-position it while still on the limb	57 (27.8%)	45 (34.6%)	2 (13.3%)	10 (16.6%)		
What minimum information must be documented in the operation notes regarding pneumatic tourniquets?					12.39	0.002
Name of the person who applied the cuff	35 (17%)	32 (24.6%)	0 (0%)	3 (5%)		
Site of cuff placement	40 (19.5%)	30 (23%)	3 (20%)	7 (11.6%)		
Type of skin protection that was applied	25 (12.1%)	18 (13.8%)	0 (0%)	7 (11.6%)		
All of the above	105 (51.2%)	40 (30.7%)	12 (80%)	43 (71.6%)		
Why is it important to prevent fluids from leaking beneath the cuff?					30.43	0.00003
To prevent the tourniquet from slipping from its place of application	61 (29.7%)	24 (18.4%)	4 (26.6%)	33 (55%)		
To prevent damage from diathermy	40 (19.5%)	33 (25.3%)	2 (13.3%)	5 (8.3%)		
To prevent chemical burns	50 (24.3%)	33 (25.3%)	7 (46.6%)	10 (16.6%)		
To prevent damage from diathermy and to prevent chemical burn	22 (10.7%)	17 (13%)	1 (6.6%)	4 (6.6%)		
To prevent the tourniquet from slipping from its place of application and to prevent chemical burns	18 (8.7%)	14 (10.7%)	0 (0%)	4 (6.6%)		
To prevent the tourniquet from slipping from its place of application and to prevent damage from diathermy	12 (5.8%)	8 (6.1%)	0 (0%)	4 (6.6%)		
All three	2 (0.9%)	1 (0.7%)	1 (6.6%)	0 (0%)		
Why must the cuff be inflated rapidly?					36.12	<0.001
Rapid inflation simultaneously compresses arteries and veins, preventing the veins from filling before the compression of the arteries	135 (65.8%)	70 (53.8%)	7 (46.6%)	58 (96.6%)		
Rapid inflation simultaneously compresses arteries and veins, preventing the arteries from filling before the compression of the veins	70 (34.1%)	60 (46.15%)	8 (53.3%)	2 (3.3%)		
Is an underlying sleeve or padding material normally used under the cuff?					14.7	0.0006
Yes	152 (74.1%)	87 (66.9%)	13 (86.6%)	55 (91.6%)		
No	53 (25.8%)	43 (33%)	2 (13.3%)	5 (8.3%)		
Which underlying sleeve or padding material is normally used under the cuff?					19.99	0.004
Cotton cast padding	140 (68.2%)	75 (57.6%)	11 (73.3%)	54 (90%)		
Stockinette	65 (31.7%)	55 (42.3%)	4 (26.6%)	6 (10%)		
How is it calibrated and maintained					17.39	0.0016
Weekly	50 (24.3%)	42 (32.3%)	2 (13.3%)	6 (10%)		
Monthly	80 (39%)	48 (36.9%)	3 (20%)	29 (48.3%)		
Yearly	75 (36.5%)	40 (30.7%)	10 (66.6%)	25 (41.6%)		

Knowledge of limb occlusion pressure (LOP), the cuff shape that occludes arterial flow at a lower pressure, contraindications for pneumatic tourniquet use, minimum information that must be documented in the operation notes regarding pneumatic tourniquets, importance of preventing fluids from leaking out beneath the cuff, importance of inflating the cuff rapidly, the underlying sleeves or padding materials under the cuffs, and how regularly the tourniquet is calibrated and maintained was found to be statistically significant, with a p-value of <0.01, 0.007, 0.0079, 0.002, 0.00003, <0.001, 0.004, and 0.0016, respectively, among the residents, consultants, and specialists (Table 2). 

**Figure 1:**
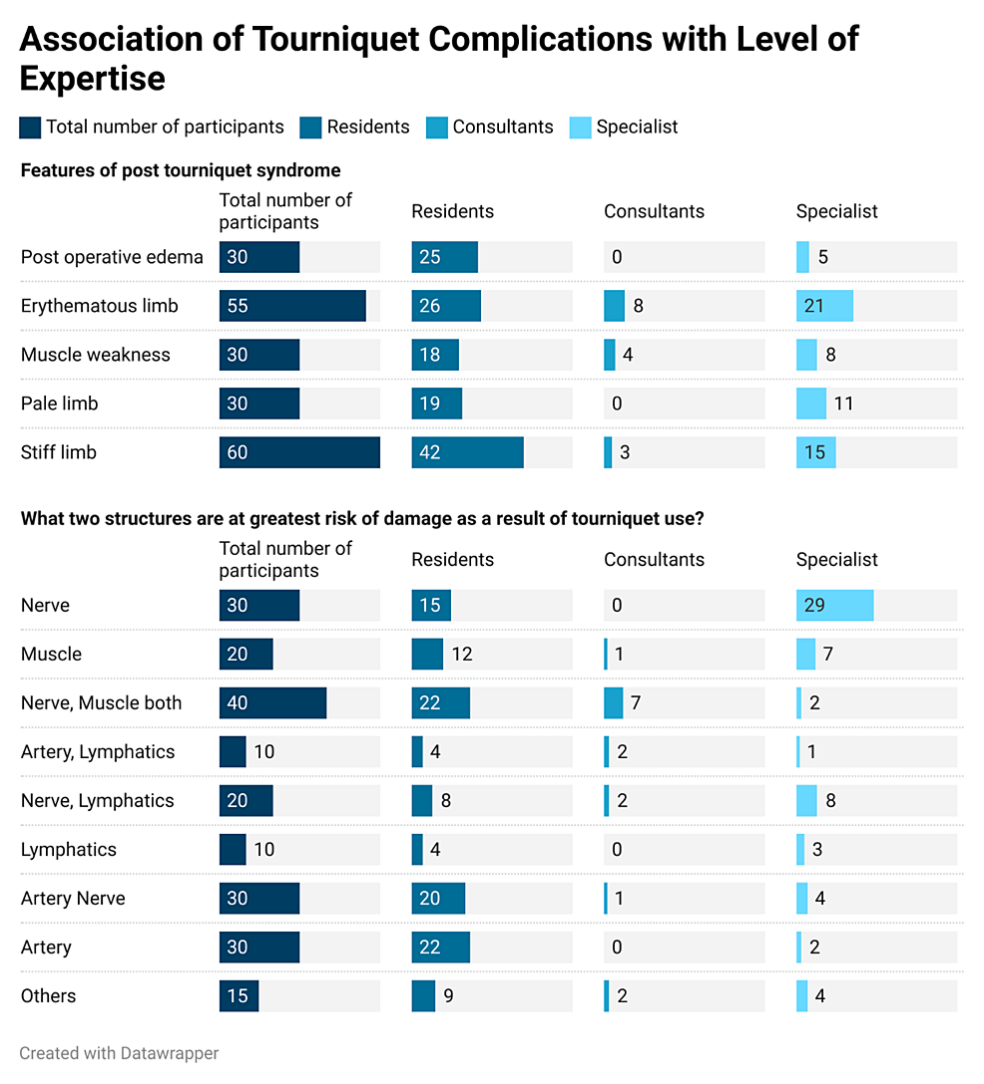
Association of tourniquet complications with the level of expertise

The correlation between post-tourniquet syndrome and the participant’s level of expertise was further investigated. We observed that the incidence of features of post-operative tourniquet syndrome was not significantly associated with the participants’ level of expertise (p=0.12). Tourniquet use affected nerves, muscles, and arteries more commonly; however, this was not significantly associated with the knowledge and experience of all groups (p=0.10) (Figure 1).

**Figure 2:**
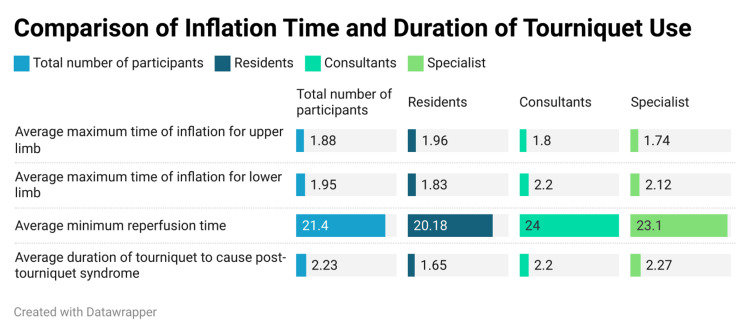
Comparison of inflation time and duration of tourniquet use

The average maximum inflation time was observed to be 1.88 ± 1.43 hours for the upper limb. We observed a slight difference in responses between the specialists and others; however, these differences were not statistically significant. The specialist group reported an average maximum inflation time of 1.74 ± 0.42 hours for the upper limb and 1.95± 0.59 hours for the lower limb. We observed a slight difference in responses between the residents and the other groups, and this was statistically significant (p=0.0006) (Figure 2).

A post-hoc analysis revealed that the residents needed lesser inflation times for the lower limbs than the consultants (p=0.004) (Table 3). The overall average minimum time of reperfusion was observed to be 21.4 ± 8.4 hours. We observed a statistically significant difference between the responses of residents and those of the other groups (p=0.04).

**Table 3:** Post-hoc analysis of maximum time of inflation for lower limb

Pairwise comparison	HSD_0.05_ = 0.292 HSD_0.01_ = 0.364	Q_0.05_ = 3.340 Q_0.01_ =4.17	p-value
Residents vs. specialist	0.29	3.29	0.05
Residents vs. consultants	0.39	4.50	0.004
Specialist vs. consultants	0.11	1.22	0.66

However, we failed to identify the differences in time of inflation between residents and consultants and between residents and specialists during the post-hoc analysis (Table 4).

**Table 4:** Post-hoc analysis of reperfusion time

Pairwise comparison	HSD_0.05_ = 4.34 HSD_0.01_ = 5.42	Q_0.05_ = 3.34 Q_0.01_ = 4.17	p-value
Residents vs. consultants	3.46	2.66	0.14
Residents vs. specialist	2.93	2.25	0.25
Consultants vs. specialist	0.53	0.41	0.9

Post-tourniquet incidents were observed within a mean duration of 2.23 ± 3.03 hours (Figure 2). The post-hoc analysis revealed that residents recorded higher post-tourniquet syndrome incidents than consultants and specialists (p=0.006 and p=0.0019) (Table 5). 

**Table 5:** A post-hoc analysis of post-tourniquet syndrome

Pairwise comparison	HSD_0.05_ = 0.53 HSD_0.01_ = 0.66	Q_0.05_ = 3.34 Q_0.01_ = 4.17	p-value
Residents vs. consultants	0.71	4.39	0.006
Residents vs. specialist	0.62	3.85	0.019
Consultants vs. specialist	0.09	0.55	0.92

## Discussion

The purpose of the study was to evaluate Saudi orthopaedics' understanding of tourniquet use, as well as the practical aspects associated with tourniquet use and the complications they encountered in their practices. In this study, a total of 205 people participated in the survey. Responses were recorded from 130 medical residents, 15 consultants and 60 specialists. Proximal to the incision on the bone was the preferred location for the cuff in 125 (60.9%) of the participants, followed by distal to the incision on the joint in 35%, proximal to the incision on the joint in 25%, and distal to the incision on the bone in 9%. The expert group observed a 1.74±0.42 average maximum inflation time for the upper limb. The lower limb's maximal inflating time was reported to be 1.95±0.59 on average.

Digital tourniquets have a monitor that displays the cuff pressure and inflation time, an inflatable cuff, a compressed gas source, and a microprocessor-controlled pressure regulator that maintains the cuff pressure [22]. Cuff leaks, unusually high or low cuff pressures, and prolonged tourniquet duration activate an audible and visual alert [22]. Because of the benefits of maintaining a blood-free operative field, tourniquets continue to be widely used by orthopaedic surgeons despite data suggesting that they are unnecessary for some types of orthopaedic surgery [8-10]. Under the close supervision of trained professionals and with the correct inflation pressures and duration of tourniquet application, tourniquet-related complications are uncommon. By administering the proper analgesia (such as opioids) and local anaesthetic agents and applying appropriate localized methods such as blocks, it is possible to reduce the incidence of complications associated with tourniquet use [12,23].

In this study, 205 responses were obtained from 130 residents, 15 consultants, and 60 specialists. Fifty (24.3%) of the respondents practised in a community hospital, while 135 (65.8%) practised in a teaching hospital. In a similar study, Boya et al. enrolled 98 participants actively working in orthopaedic units; most of them worked in a university hospital (n=48, 49%) [24].

The optimal tourniquet CP still needs to be determined [8,12]. A common practice among orthopaedic surgeons is inflating cuffs to pressures that are higher than necessary [8]. Recommendations for LOP, artery occlusion pressure, and Doppler occlusion pressure, as well as the use of CPs twice the systolic blood pressure, calculating CPs using an equation, and adding a safety margin to the systolic blood pressure, have emerged in recent years [8,12,25,26].

The most prevalent adverse response to tourniquet use was pain. There was a strong correlation between the duration of tourniquet use and the frequency and severity of pain. Therefore, tourniquets should be used sparingly, if at all. In the event of tourniquet-related complications, the patient may encounter significant medical and legal consequences [10,27,28]. Most surgical trainees need to be formally instructed on tourniquet use, and consequently, most surgeons need to be taught how to correctly use them. Moreover, the law mandates that every six months, all employees must complete a remedial course on tourniquet use [29].

Surgeons’ use of tourniquets is rarely discussed in the medical literature. The use of tourniquets is rising, and this was investigated by emailing a survey to orthopaedic surgeons in North America; it was found that 2.5% of the participants employed the use of an Esmarch bandage tourniquet at the ankle, whereas 3.4% never or rarely used a tourniquet [18]. Most (92%; 27%) of the respondents applied pneumatic cuffs to the ankle, 69% applied them to the thighs, and 15% applied them to the calves. Sixty-two per cent of individuals who used thigh cuffs reported experiencing occasional or frequent difficulties with cuff fit. Almost always (97%), the limb was exsanguinated before the tourniquet was administered. For exsanguination, surgeons utilized a variety of instruments; 64% of them applied pressures between 300 and 350 mmHg when applying tourniquets to the ankle and quadriceps. Only 7% of respondents considered LOP when choosing the tourniquet CP [18]. This finding is similar to that in our study.

Based on established protocol, tourniquet application is typically delegated to a specific staff member (a security guard, nurse, or surgeon's assistant) [29]. Inflation and deflation of tourniquets are associated with systemic issues. In contrast, cuff compression and tissue hypoperfusion directly impact the local region [10,22]. The most frequently reported side effect is blistering of the epidermis, along with muscle and nerve damage. The combination of the tourniquet's mechanical compression and the protracted ischemia causes neuromuscular difficulties [10,20]. Under the tourniquets, chemical burns may occur if the cast material is saturated with skin-preparation solutions. These results suggest that residents and orthopaedic surgeons may have preconceived notions regarding this topic. Despite being aware of tourniquet-related complications, orthopaedic surgeons may need to pay more attention to them due to the high-risk nature of their profession.

Although they must be renewable and preferences based on them must be congruent with scientific facts, life experiences have a worth that cannot be discounted. This study's objectives were to investigate the knowledge of tourniquet use among orthopaedic surgeons in Saudi and to evaluate the practical aspects of their use of tourniquets and the complications they have experienced in their practices.

Limitation

The study being conducted online, and the small sample size can influence the generalizability of study findings.

## Conclusions

When used properly, tourniquets are invaluable for limb surgeries as they prevent excessive bleeding and keep the surgical field clean. The findings of this survey should serve as a reminder to the orthopedic community that it is time to reevaluate long-standing practices, particularly about the optimal tourniquet pressure. The addition of ligature safety information to the current orthopedic curriculum and outlining the settings and procedures for applying pressure should also be considered. It is vital to prevent complications by establishing a standard cuff pressure and procedure standards. This study highlighted the significance of modifying the training of residents to increase knowledge and awareness and reduce the occurrence of complications. More data are needed in Saudi Arabia, and further studies are required.

## References

[1] Odinsson A, Finsen V (2006). Tourniquet use and its complications in Norway. J Bone Joint Surg Br.

[2] Noordin S, McEwen JA, Kragh JF Jr, Eisen A, Masri BA (2009). Surgical tourniquets in orthopaedics. J Bone Joint Surg Am.

[3] Eidelman M, Katzman A, Bialik V (2006). A novel elastic exsanguination tourniquet as an alternative to the pneumatic cuff in pediatric orthopedic limb surgery. J Pediatr Orthop B.

[4] Boiko M, Roffman M (2004). Evaluation of a novel tourniquet device for bloodless surgery of the hand. J Hand Surg Br.

[5] Klenerman L (1962). The tourniquet in surgery. J Bone Joint Surg Br.

[6] Papalia R, Zampogna B, Franceschi F, Torre G, Maffulli N, Denaro V (2014). Tourniquet in knee surgery. Br Med Bull.

[7] Grover C, Nanda S, Nagi Reddy BS (2014). Gauze strip tourniquet for nail surgery. J Cutan Aesthet Surg.

[8] Fitzgibbons PG, Digiovanni C, Hares S, Akelman E (2012). Safe tourniquet use: a review of the evidence. J Am Acad Orthop Surg.

[9] Kam PC, Kavanagh R, Yoong FF (2001). The arterial tourniquet: pathophysiological consequences and anaesthetic implications. Anaesthesia.

[10] Cox C, Yao J (2010). Tourniquet usage in upper extremity surgery. J Hand Surg Am.

[11] Aziz ES (2009). Tourniquet use in orthopaedic anesthesia. Curr Anaesth Crit Care.

[12] Sharma JP, Salhotra R (2012). Tourniquets in orthopedic surgery. Indian J Orthop.

[13] Younger AS, Kalla TP, McEwen JA, Inkpen K (2005). Survey of tourniquet use in orthopaedic foot and ankle surgery. Foot Ankle Int.

[14] Yang JH, Lim H, Yoon JR, Jeong HI (2012). Tourniquet associated chemical burn. Indian J Orthop.

[15] Kumar K, Railton C, Tawfic Q (2016). Tourniquet application during anesthesia: "What we need to know?". J Anaesthesiol Clin Pharmacol.

[16] Morrison RS, Magaziner J, McLaughlin MA (2003). The impact of post-operative pain on outcomes following hip fracture. Pain.

[17] Ducic I, Chang S, Dellon AL (2006). Use of the tourniquet in reconstructive surgery in patients with previous ipsilateral lower extremity revascularization: is it safe? A survey. J Reconstr Microsurg.

[18] Kalla TP, Younger A, McEwen JA, Inkpen K (2003). Survey of tourniquet use in podiatric surgery. J Foot Ankle Surg.

[19] AORN Recommended Practices Committee (2007). Recommended practices for the use of the pneumatic tourniquet in the perioperative practice setting. AORN J.

[20] Yalçınkaya M, Sökücü S, Erdoğan S, Kabukçuoğlu YS (2014). Tourniquet use in orthopedic surgery: a descriptive survey study among Turkish orthopedic surgeons and residents in Istanbul. Acta Orthop Traumatol Turc.

[21] Asghar A, Narayan RK, Kumar A, Naaz S (2020). The transcortical vessel is replacement of cortical capillary or a separate identity in diaphyseal vascularity. Anat Cell Biol.

[22] Asghar A, Kumar A, Kant Narayan R, Naaz S (2020). Is the cortical capillary renamed as the transcortical vessel in diaphyseal vascularity?. Anat Rec (Hoboken).

[23] Saied A, Ayatollahi Mousavi A, Arabnejad F, Ahmadzadeh Heshmati A (2015). Tourniquet in surgery of the limbs: a review of history, types and complications. Iran Red Crescent Med J.

[24] Boya H, Tuncalı B, Özcan Ö, Araç Ş, Tuncay C (2016). Practice of tourniquet use in Turkey: a pilot study. Acta Orthop Traumatol Turc.

[25] Tejwani NC, Immerman I, Achan P, Egol KA, McLaurin T (2006). Tourniquet cuff pressure: the gulf between science and practice. J Trauma.

[26] Tuncali B, Karci A, Tuncali BE (2006). A new method for estimating arterial occlusion pressure in optimizing pneumatic tourniquet inflation pressure. Anesth Analg.

[27] Sadri A, Braithwaite IJ, Abdul-Jabar HB, Sarraf KM (2010). Understanding of intra-operative tourniquets amongst orthopaedic surgeons and theatre staff--a questionnaire study. Ann R Coll Surg Engl.

[28] Klenerman L (2003). The tourniquet manual: principles and practice. https://link.springer.com/book/10.1007/b97532.

[29] Daruwalla ZJ, Rowan F, Finnegan M, Fennell J, Neligan M (2012). Exsanguinators and tourniquets: do we need to change our practice?. Surgeon.

